# USING CO-DESIGN TO DEVELOP A SCHOOL-BASED ORAL HYGIENE INTERVENTION LINKING YOUTH AND OLDER ADULTS

**DOI:** 10.1093/geroni/igae098.1629

**Published:** 2024-12-31

**Authors:** Lingyi Shen, Liangyi Jin, Hanzhang Xu

**Affiliations:** Duke Kunshan University, Kunshan, Jiangsu, China (People’s Republic); Duke Kunshan University, Kunshan, Jiangsu, China (People’s Republic); Duke University, Durham, North Carolina, United States

## Abstract

The prevalence of oral health problems is high among Chinese older adults, which was attributed to limited oral health knowledge and poor oral hygiene behaviors. One key strategy to achieve optimal oral health is through health education. School settings are ideal to disseminate and implement public health interventions to a large group at a relatively low cost. As more than half of the Chinese older adults provide care to at least one grandchild, mobilizing these strong, existing intergenerational ties that Chinese older adults share has great potential to enhance the effect of school-based oral health promotion programs across generations. This study aimed to describe an older adult-grandchild co-design process that will lead to the development of a school-based oral health education intervention to improve oral hygiene behavior for both youth and older adults. A mixed-methods two-step sequential design was used. Step 1: focus groups among older adults (n=12), their grandchildren (n=12), and elementary school teachers (n=8) were conducted and analyzed to describe their knowledge and attitude towards oral hygiene behaviors. Step 2: vignettes were designed based on focus group results and presented to older adults, their grandchildren (5 dyads) and 2 elementary school teachers to co-design interventions to address needs. Three key themes were identified: perceived importance, power dynamic in the family, and limited time and resources. A few potential intervention strategies were produced, including leveraging school assignments to promote oral hygiene behaviors, building a rewarding mechanism, enhancing teacher-grandparents communications, and building oral health awareness in the community.

